# *In ovo* feeding of probiotic lactobacilli differentially alters expression of genes involved in the development and immunological maturation of bursa of Fabricius in pre-hatched chicks

**DOI:** 10.1016/j.psj.2023.103237

**Published:** 2023-10-25

**Authors:** Shreeya Sharma, Raveendra R. Kulkarni, Shayan Sharif, Hosni Hassan, Mohammadali Alizadeh, Scott Pratt, Khaled Abdelaziz

**Affiliations:** ⁎ Department of Animal and Veterinary Sciences, College of Agriculture, Forestry and Life Sciences, Clemson University, Clemson, SC, USA; † Department of Population Health and Pathobiology, North Carolina State University, Raleigh, NC, USA; ‡ Department of Pathobiology, Ontario Veterinary College, University of Guelph, Guelph, Ontario N1G 2W1, Canada; § Prestage Department of Poultry Science, North Carolina State University, Raleigh, NC, USA

**Keywords:** chicken, probiotics, *Lactobacillus*, cytokine, bursa of Fabricius

## Abstract

Compelling evidence indicates that immunological maturation of the gut-associated lymphoid tissues, including the bursa of Fabricius, is dependent upon antigenic stimulation post-hatch. In view of these data, the present study investigated the impact of exposing the immune system of chick embryos to antigenic stimuli, via *in ovo* delivery of poultry-specific lactobacilli, on the expression of genes associated with early bursal development and maturation. Broiler line embryonated eggs were inoculated with 10^6^ and 10^7^ colony-forming units (**CFUs**) of an individual or a mixture of *Lactobacillus* species, including *L. crispatus* (C25), *L. animalis* (P38), *L. acidophilus* (P42), and *L. reuteri* (P43), at embryonic day 18 (**ED18**). The bursa of Fabricius was collected from pre-hatched chicks (ED20) to measure the expression levels of various immune system genes. The results revealed that *L. acidophilus* and the mixture of *Lactobacillus* species at the dose of 10^6^ CFU consistently elicited higher expression of genes responsible for B cell development, differentiation, and survival (B cell activating factor (**BAFF**), BAFF-receptor (**BAFF-R**)), and antibody production (interleukin (IL)-10) and diversification (TGF-β). Similar expression patterns were also noted in T helper (**Th**) cell-associated cytokine genes, including Th1-type cytokines (interferon (**IFN**)-γ and IL-12p40), Th2-type cytokines (IL-4 and IL-13) and Th17 cytokine (IL-17). Overall, these results suggest that the supplementation of poultry-specific lactobacilli to chick embryos might be beneficial for accelerating the development and immunological maturation of the bursa of Fabricius. However, further studies are required to determine if the changes in gene expression are associated with the developmental trajectory and phenotypes of bursal cells.

## INTRODUCTION

Studies indicate that the gut immune system in chickens is not fully developed at hatch; it begins to develop during early embryogenesis and gradually matures during the first few weeks after hatching (Lowenthal et al., 1994; Bar-Shira et al., 2003; Miyazaki et al., 2007). The gut immune system is mainly orchestrated by the gut-associated lymphoid tissue (**GALT**), the largest immune organ in chickens. GALT is a complex network of innate and adaptive immune cells that function together to maintain gut-immunological homeostasis (Mowat and Agace, 2014) and includes the bursa of Fabricius, Peyer's patches, cecal tonsils, Meckel's diverticulum, and intraepithelial and lamina propria lymphocytes (Smith et al., 2014; Taha-Abdelaziz et al., 2018).

The bursa of Fabricius is the largest GALT organ and possesses a unique microenvironment supporting the development of mature B cells. Although the bursa of Fabricius contains a small population of thymus-derived lymphocytes (T cells), there is substantial evidence highlighting their crucial role in providing protection against viral diseases, such as infectious bursal disease and Marek's disease (Kim et al., 2000; Schat, 2022). The maturation and differentiation of the B cells in the bursa is a highly regulated process that relies on early exposure to antigens after hatching (Glick et al., 1956; Sorvari et al., 1975); however, modern poultry farming practices, including egg decontamination, artificial incubation, hatchery sanitation, lack of exposure to maternal microbiota, and the prophylactic use of antibiotics, were associated with a delay in the bursal development (Kpodo and Proszkowiec-Weglarz, 2023). The delay in bursal maturation renders hatched chicks susceptible to a plethora of environmental pathogens.

Experimental evidence indicates that early post-hatch feeding supplies the antigens required for the differentiation and proliferation of B lymphocytes in the GALT, including the bursa of Fabricius (Nagler-Anderson, 2001; Taha-Abdelaziz et al., 2018; Reicher et al., 2022). Additionally, recent data suggests that supplying antigens to chick embryos before hatching could result in outcomes comparable to post-hatch application. This has paved a new avenue for research into exploring the potential ability of *in ovo* delivery of feed additives, including probiotics, to chick embryos in eliciting early immunity and enhancing the immune competence of hatched chicks (Kadam et al., 2013; Madej et al., 2015; Teague et al., 2017). For instance, *in ovo* administration of probiotic lactobacilli has been shown to induce expression of immune-related genes in lymphoid organs and improve systemic antibody response to different antigens, such as sheep red blood cells (**SRBC**) and keyhole limpet hemocyanin (**KLH**) in newly hatched chicks, despite the incomplete development of their immune system (Alizadeh et al., 2020, 2021, 2022). In another study, supplementation of *Lactobacillus* species to chick embryos was shown to enhance cellular proliferation and increase the weight of lymphoid organs, including the spleen, thymus, and bursa of Fabricius, suggesting a possible role for probiotics in modulating the development of these lymphoid organs (Madej et al., 2015). It is important to emphasize that probiotic lactobacilli isolated and reinoculated into the same host species (i.e., species-specific lactobacilli) demonstrated greater effectiveness compared to those isolated from different species (La Reau et al., 2016; Kido et al., 2021, Moeller et al., 2019). However, although much research focused on the effects of *in ovo*-delivered probiotics on immune responses and cellular and morphological changes in lymphoid organs of hatched chicks, there remains no clear evidence of whether lactobacilli influence the development and functional maturation of these organs in pre-hatched chicks at the molecular level.

Considering the role of the bursa of Fabricius, the primary site for the development of the B cell repertoire, in antibody production and in providing a niche for other lymphoid and myeloid cells such as T cells, macrophages, and dendritic cells, this study was undertaken to investigate the potential role of poultry-specific lactobacilli in modulating the expression of genes involved in the development, differentiation and activation of B cells and functional activity of other bursal cells, in pre-hatched chicks.

## MATERIALS AND METHODS

**Eggs Incubation**

Eighty-eight embryonated commercial Ross 308 broiler eggs were obtained from a commercial hatchery (Fieldale Farms Corporation, GA) and incubated in an isolated, disease-free facility/Biosecurity Level-2 laboratory in a sanitized egg incubator (GQF Manufacturing Company Inc., GA) set at 37°C with humidity at 55% and automated turning for 20 d. All procedures were approved by the Institutional Animal Care and Use Committee (**IACUC**) at Clemson University.

### Probiotic Strains

Poultry-specific *Lactobacillus* strains (*L. reuteri*-P43, *L. acidophilus*-P42, *L. animalis*-P38, and *L. crispatus*-C25 were provided by H. M. Hassan's Laboratory at NC State University). The growth curve for each strain utilized in this study was generated by inoculating a loopful of frozen *Lactobacillus* species into a 14 mL bacterial culture tube containing 10 mL of MRS (DeMan, Rogosa, and Sharpe). Tubes were incubated overnight at 37°C under anaerobic conditions. Subsequently, 500 μL of the overnight culture was inoculated into 50 mL fresh MRS broth and incubated at 37°C under anaerobic conditions. The optical density (**OD**) of each culture was measured at 600 nm (**OD600**) using a spectrophotometer (VWR, PA) at various time intervals. To determine the number of colony-forming units (**CFUs**) of each *Lactobacillus* strain/mL, a 10-fold serial dilution of each growing culture was prepared, and 100 μL of each dilution was streaked onto MRS agar plates. The plates were then incubated for 24 h at 37°C under anaerobic conditions. The viable colony counts were enumerated, and a linear calibration curve was generated by plotting CFUs/mL against the corresponding OD measurements obtained at different time points. A linear regression analysis was then conducted, and individual growth formulas were generated for each *Lactobacillus* strain to transform ODs into viable counts. The *Lactobacillus* strains used for *in ovo* injection were grown individually in MRS broth and incubated at 37°C under anaerobic conditions. During the stationary phase (15 h of incubation), individual *Lactobacillus* suspension was adjusted to a concentration of 10^6^ or 10^7^ CFUs/100 μL of PBS, and equal concentrations of each culture were mixed together to prepare a multistrain cocktail of lactobacilli.

### *In ovo* Inoculations

On embryonic day 18 (**ED18**), eggs were disinfected with 70% ethanol and were randomly divided into 11 treatment groups with 8 replicates each. Subsequently, a hole was punched in the eggshell and a 23-gauge needle was used to administer the lactobacilli into the amniotic sac. Eggs were injected with different doses (low: 10^6^ CFU or high: 10^7^ CFU) of the individual *Lactobacillus* species or their mixture in a total volume of 100 μL Dulbecco's Phosphate-Buffered Saline (**DPBS**). The 11th group was treated with DPBS and served as a negative control. The treatment groups are summarized in Table 1. Eggs were incubated at 37°C with humidity at 55% and automated turning. After 48 h post-inoculation (ED20), chick embryos were sacrificed, and the bursa of Fabricius was collected in 1 mL TRIzol (Invitrogen, Carlsbad, CA) and stored at −80°C until further processing.

**Table 1:** Treatment groups.

Treatment group	*Lactobacillus* strain	Dose (CFUs)	Necropsy day	Tissue collected
1	*L. animalis*	10^6^	ED20	Bursa of Fabricius
2	*L. animalis*	10^7^		
3	*L. acidophilus*	10^6^		
4	*L. acidophilus*	10^7^		
5	*L. reuteri*	10^6^		
6	*L. reuteri*	10^7^		
7	*L. crispatus*	10^6^		
8	*L. crispatus*	10^7^		
9	Mixture	10^6^		
10	Mixture	10^7^		
11	PBS	-		

### RNA Extraction and Complementary DNA (cDNA) Synthesis

Tissue samples were homogenized for RNA extraction using Bead Ruptor Elite (Omni International, GA). RNA was extracted from the bursa of Fabricius using TRIzol (Invitrogen, Carlsbad, CA) per the manufacturer's protocol. Total RNA was treated with DNase (DNA-free kit, Invitrogen, Carlsbad, CA) to remove the genomic DNA. The mass and purity of RNA samples were measured by a Nanodrop One spectrophotometer (Thermo Scientific, Greenville County, SC). Reverse transcription to cDNA was performed using SuperscriptII First-Strand Synthesis kit (Invitrogen, Carlsbad, CA) and oligo-dT primers (Thermofisher Scientific, Greenville County, SC) according to the manufacturer's protocol. The cDNA was diluted 1:10 in nuclease-free water.

### Quantitative Real-Time PCR

Quantitative real-time PCR (**qRT-PCR**) was performed using the LightCycler480 system (Roche Diagnostics) as previously described (Taha-Abdelaziz et al., 2016). The PCR master mix consisted of 10 µL of PowerTrack SYBR Green Master Mix (ThermoFisher Scientific, Greenville County, SC), 1 µL of forward and 1 µL of reverse primers (10 µM) and 3 µL nuclease-free water. Each reaction consisted of 15 µL of the master mix and 5 uL of cDNA. The qRT-PCR cycling protocol included an initial denaturation step at 95°C, followed by amplification for 45 cycles consisting of 95°C for 10 s, annealing (according to specific primers; provided in Table 2), and extension at 72°C for 10 s. All primers used in this study (Table 2) were synthesized by MilliporeSigma (Burlington, MA). The expression of the target genes was calculated relative to the reference gene (β-actin) using the Roche LightCycler 480 software based on the 2^−ΔΔCT^ method.

**Table 2:** Primer sequences used for real-time quantitative PCR.

Gene	Primer sequence (5′–3′)	Annealing temp. (°C)	References
β-actin	F: CAACACAGTGCTGTCTGGTGGTA R: ATCGTACTCCTGCTTGCTGATCC	60	Brisbin et al. (2010)
IFN-γ	F: ACACTGACAAGTCAAAGCCGCACA R: AGTCGTTCATCGGGAGCTTGGC	60	Brisbin et al. (2010)
IL-10	F: TTTGGCTGCCAGTCTGTGTC R: CTCATCCATCTTCTCGAACGTC	64	Taha-Abdelaziz et al. (2017)
IL-17	F: TATCAGCAAACGCTCACTGG R: AGTTCACGCACCTGGAATG	60	Crhanova et al. (2011)
IL-13	F: ACTTGTCCAAGCTGAAGCTGTC R: TCTTGCAGTCGGTCATGTTGTC	60	Taha-Abdelaziz et al. (2018)
IL-4	F: GCTCTCAGTGCCGCTGATG R: GGAAACCTCTCCCTGGATGTC	58	Slawinska et al. (2014)
IL-12p40	F: TTGCCGAAGAGCACCAGCCG R: CGGTGTGCTCCAGGTCTTGGG	64	Brisbin et al. (2010)
BAFF	F: CACGTCATCCAGCAGAAGGAT R: ACAAGAGGACAGGAGCATTGC	55	Ko et al. (2018)
BAFF-R	F: CCTGGCCCCACCATAAGG R: CATTACAGTCTCTCCTCACCCATACA	55	Ko et al. (2018)
TGF-β	F: CGGCCGACGATGAGTGGCTC R: CGGGGCCCATCTCACAGGGA	60	Taha-Abdelaziz et al. (2018)

### Statistical Analysis

Data were analyzed and graphs were created using GraphPad Prism V5.0 (GraphPad Software, San Diego, CA). The effects of lactobacilli on cytokine expression were analyzed using 1-way ANOVA and differences among means between the treatment groups were determined using Tukey's multiple comparison test. Results were considered statistically significant if the *P* value ≤ 0.05. Data are shown graphically as the mean of the relative gene expression data (2^−∆∆Ct^) ± the standard error of the mean (**SEM**).

## RESULTS

### Effects of *In ovo*-Delivered Lactobacilli on the Gene Expression of Cytokines Involved in B Cell Development, Differentiation, Activation, and Survival in the Bursa of Fabricius

**Figure 1:**
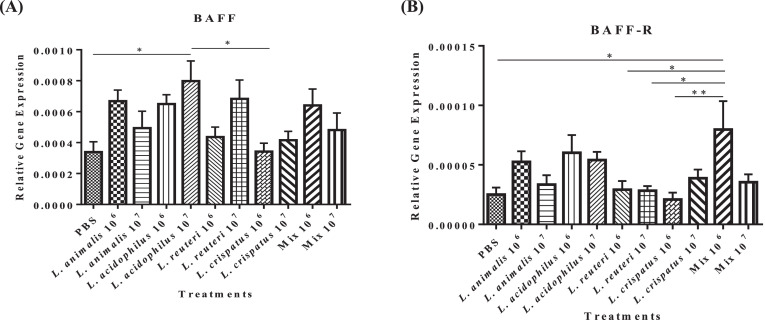
Effect of *in ovo* supplementation of different *Lactobacillus* species on the relative gene expression profiles of cytokines involved in B cell development, differentiation, activation, and survival: BAFF (A) and BAFF-R (B) in the bursa of pre-hatched chick embryos. On embryonic d 18, eggs were injected with the respective *Lactobacillus* treatments and the bursa of Fabricius was collected at 48 h post-treatment. The treatment groups were as follows: PBS (phosphate-buffered saline), *L. animalis* (10^6^ CFU/egg), *L. animalis* (10^7^ CFU/egg), *L. acidophilus* (10^6^ CFU/egg), *L. acidophilus* (10^7^ CFU/egg), *L. reuteri* (10^6^ CFU/egg), *L. reuteri* (10^7^ CFU/egg), *L. crispatus* (10^6^ CFU/egg), *L. crispatus* (10^7^ CFU/egg), and a mixture of the lactobacilli at the dose of 10^6^ CFU/egg and 10^7^ CFU/egg. The expression of the target genes was calculated relative to the reference gene (β-actin). Statistical significance among treatment groups was calculated using 1-way ANOVA followed by Tukey's comparison test. Error bars represent the standard error of the mean (SEM). An asterisk indicates a significant difference (*P* ≤ 0.05) among the treatment groups.

The high dose of *L. acidophilus* (10^7^ CFUs) significantly induced a higher expression (*P* ≤ 0.05) of the B cell-activating factor (**BAFF**; an essential element for B cell maturation, differentiation, and survival) (Ng et al., 2004) than the untreated control and *L. crispatus* (10^6^ CFU) treated group. A numerically higher expression of BAFF was observed in *L. animalis* (10^6^ CFU), *L. acidophilus* (10^6^ CFU), *L. reuteri* (10^6^ CFU), and *Lactobacillus* mixture (10^6^ CFU) treatment groups compared to the untreated control group (Figure 1A).

With respect to BAFF-receptor (**BAFF-R**), a critical regulator of B cell function, *L. animalis* (10^6^ CFU) and *L. acidophilus* at both low and high doses (10^6^ and 10^7^ CFUs) induced numerically higher expression of BAFF-R in the bursa (*P* ≥ 0.05), whereas *Lactobacillus* mixture at 10^6^ CFU induced significantly higher expression of BAFF-R (*P* ≤ 0.05) than the untreated control, both low and high doses of *L. reuteri* (10^6^ and 10^7^ CFU), and a low dose of *L. crispatus* (10^6^ CFU) (Figure 1B).

**Figure 2:**
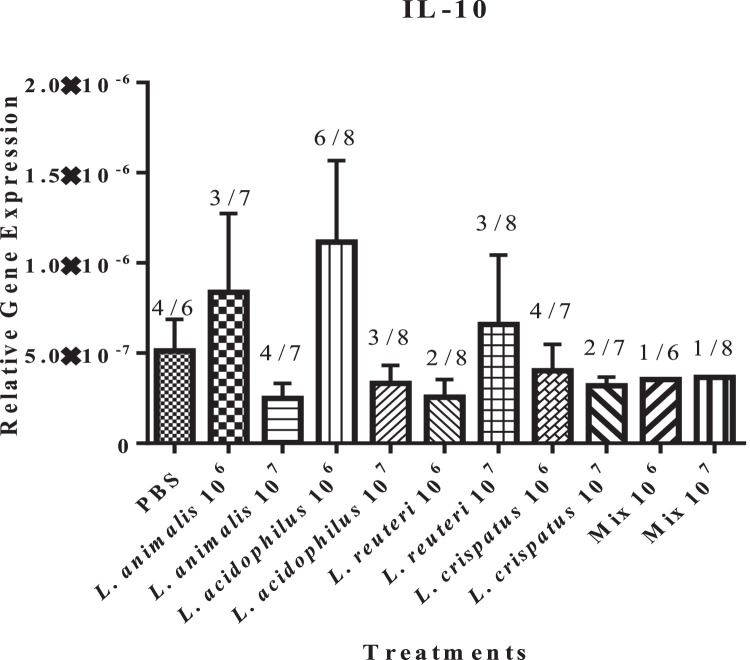
Effect of *in ovo* supplementation of different probiotic treatments on the relative gene expression profiles of the immunoregulatory pleiotropic cytokine involved in B cell activation and antibody production: IL-10 (Figure 2) in the bursa of pre-hatched chick embryos. On embryonic d 18, eggs were injected with the respective *Lactobacillus* treatments and the bursa of Fabricius was collected at 48 h post-treatment. The treatment groups were as follows: PBS (phosphate-buffered saline), *L. animalis* (10^6^ CFU/egg), *L. animalis* (10^7^ CFU/egg), *L. acidophilus* (10^6^ CFU/egg), *L. acidophilus* (10^7^ CFU/egg), *L. reuteri* (10^6^ CFU/egg), *L. reuteri* (10^7^ CFU/egg), *L. crispatus* (10^6^ CFU/egg), *L. crispatus* (10^7^ CFU/egg), and a mixture of the lactobacilli at the dose of 10^6^ CFU/egg and 10^7^ CFU/egg. The expression of the target genes was calculated relative to the reference gene (β-actin). Statistical significance among treatment groups was calculated using 1-way ANOVA followed by Tukey's comparison test. Error bars represent the standard error of the mean (SEM). The figures above the bars represent the number of birds expressing IL-10 within each respective group.

Interleukin (**IL**)-10 is a Th2 cytokine that promotes B cell activation and antibody production (Saxena et al., 2015). While the expression of IL-10 was observed in the bursa of only 4 out of 6 birds in the untreated control group and in similar or fewer numbers of lactobacilli-treated groups, the low dose (10^6^ CFUs) of *L. acidophilus* induced its expression in 6 out of 8 birds (Figure 2). Although the data did not subject to statistical analysis, the expression of IL-10 appears to be numerically higher in the group treated with the low dose of *L. acidophilus* (*P* ≥ 0.05).

**Figure 3:**
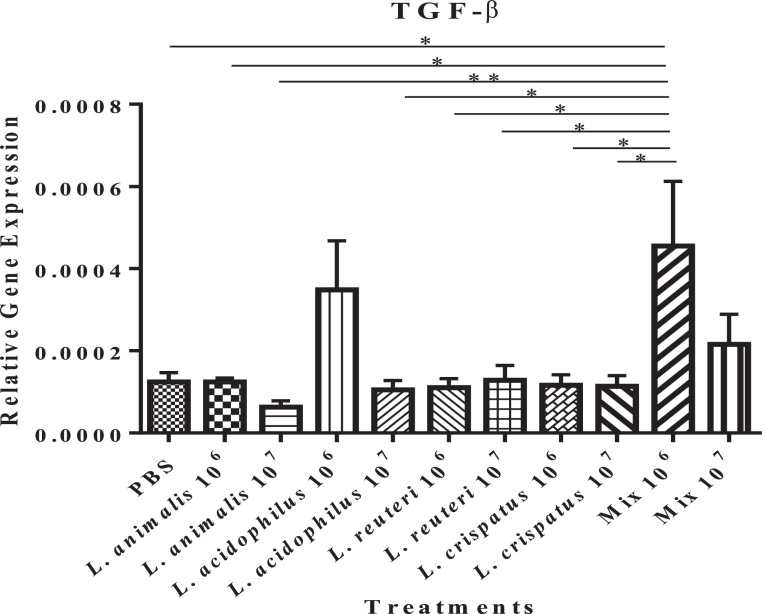
Effect of *in ovo* supplementation of different probiotic treatments on the relative gene expression profiles of the cytokine involved in antibody diversification: TGF-β (Figure 3) in the bursa of pre-hatched chick embryos. On embryonic day 18, eggs were injected with the respective *Lactobacillus* treatments and the bursa of Fabricius was collected at 48 h post-treatment. The treatment groups were as follows: PBS (phosphate-buffered saline), *L. animalis* (10^6^ CFU/egg), *L. animalis* (10^7^ CFU/egg), *L. acidophilus* (10^6^ CFU/egg), *L. acidophilus* (10^7^ CFU/egg), *L. reuteri* (10^6^ CFU/egg), *L. reuteri* (10^7^ CFU/egg), *L. crispatus* (10^6^ CFU/egg), *L. crispatus* (10^7^ CFU/egg) and a mixture of the lactobacilli at the dose of 10^6^ CFU/egg and 10^7^ CFU/egg. The expression of the target genes was calculated relative to the reference gene (β-actin). Statistical significance among treatment groups was calculated using 1-way ANOVA followed by Tukey's comparison test. Error bars represent the standard error of the mean (SEM). An asterisk indicates a significant difference (*P* ≤ 0.05) among the treatment groups.

The low dose (10^6^ CFU) of *Lactobacillus* mixture induced significantly higher expression of transforming growth factor (**TGF**)-β (a regulatory cytokine produced by T cells and other nonlymphoid cells and plays a vital role in antibody diversification) *P* ≤ 0.05) compared to the untreated control group and other treatment groups, except the low dose (10^6^ CFU) of *L. acidophilus* and the high dose of *Lactobacillus* mixture group (*P* ≥ 0.05). A numerically higher TGF-β expression was observed in the group administered with 10^6^ CFUs of *L. acidophilus* (*P* ≥ 0.05) (Figure 3) compared to the untreated control group.

### Effects of *In ovo*-Delivered Lactobacilli on the Gene Expression of T Helper (Th) Cell-Associated Cytokines in the Bursa of Fabricius

**Figure 4:**
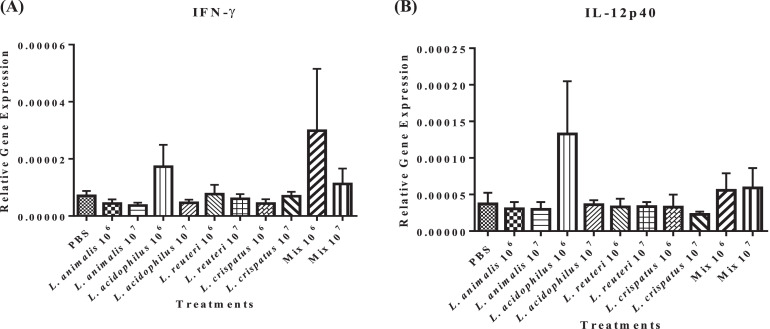
Effect of *in ovo* supplementation of different lactobacilli treatments on the relative gene expression profiles of cytokines involved in Th-1 response: IFN-γ (A) and IL-12p40 (B) in the bursa of pre-hatched chick embryos. On embryonic d 18, eggs were injected with the respective *Lactobacillus* treatments and the bursa of Fabricius was collected at 48 h post-treatment. The treatment groups were as follows: PBS (phosphate-buffered saline), *L. animalis* (10^6^ CFU/egg), *L. animalis* (10^7^ CFU/egg), *L. acidophilus* (10^6^ CFU/egg), *L. acidophilus* (10^7^ CFU/egg), *L. reuteri* (10^6^ CFU/egg), *L. reuteri* (10^7^ CFU/egg), *L. crispatus* (10^6^ CFU/egg), *L. crispatus* (10^7^ CFU/egg) and a mixture of the lactobacilli at the dose of 10^6^ CFU/egg and 10^7^ CFU/egg. The expression of the target genes was calculated relative to the reference gene (β-actin). Statistical significance among treatment groups was calculated using 1-way ANOVA followed by Tukey's comparison test. Error bars represent the standard error of the mean (SEM).

The transcriptional response of IL-12p40 (a cytokine produced by Th1 and myeloid cells, including macrophages and dendritic cells) and interferon (**IFN**)-γ (a marker of Th1-mediated immune response) (Zhu et al., 2010) were measured in the bursa of chick embryos following exposure to different doses of lactobacilli. Inoculation of 10^6^ CFUs of *L. acidophilus* and the lactobacilli mixture induced numerically higher expression of IFN-γ (*P* ≥ 0.05) compared to the untreated and other lactobacilli-treated groups (Figure 4A). The same dose of *L. acidophilus* also induced numerically higher expression of IL-12p40 (*P* ≥ 0.05) compared to the other treatment groups (Figure 4B). There were no differences among the other treatment groups in the expression of IFN-γ and IL-12p40 (*P* ≥ 0.05).

**Figure 5:**
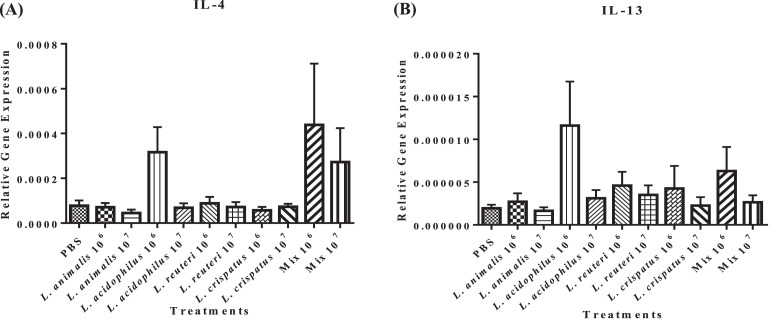
Effect of *in ovo* supplementation of different probiotic treatments on the relative gene expression profiles of cytokines involved in regulating Th-2 response: IL-4 (A) and IL-13 (B) in the bursa of pre-hatched chick embryos. On embryonic d 18, eggs were injected with the respective *Lactobacillus* treatments and the bursa of Fabricius was collected at 48 h post-treatment. The treatment groups were as follows: PBS (phosphate-buffered saline), *L. animalis* (10^6^ CFU/egg), *L. animalis* (10^7^ CFU/egg), *L. acidophilus* (10^6^ CFU/egg), *L. acidophilus* (10^7^ CFU/egg), *L. reuteri* (10^6^ CFU/egg), *L. reuteri* (10^7^ CFU/egg), *L. crispatus* (10^6^ CFU/egg), *L. crispatus* (10^7^ CFU/egg) and a mixture of the lactobacilli at the dose of 10^6^ CFU/egg and 10^7^ CFU/egg. The expression of the target genes was calculated relative to the reference gene (β-actin). Statistical significance among treatment groups was calculated using 1-way ANOVA followed by Tukey's comparison test. Error bars represent the standard error of the mean (SEM).

*In ovo* inoculation of 10^6^ CFUs of *L. acidophilus* or *Lactobacillus* mixture resulted in a numerically higher expression of IL-4 (Figure 5A) and IL-13 (Figure 5B), key regulators of Th2 response, compared to the untreated control (*P* ≥ 0.05). No differences were observed among the other treatment groups (*P* ≥ 0.05).

**Figure 6:**
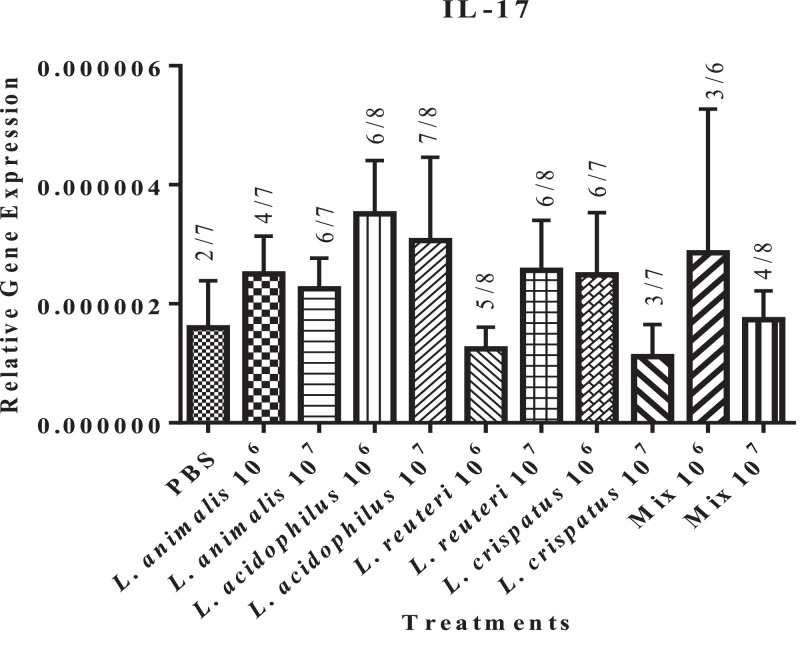
Effect of *in ovo* supplementation of different lactobacilli treatments on the relative gene expression profiles of the cytokine produced by Th-17 cell: IL-17 (Figure 6) in the bursa of pre-hatched chick embryos. On embryonic d 18, eggs were injected with the respective *Lactobacillus* treatments and the bursa of Fabricius was collected at 48 h post-treatment. The treatment groups were as follows: PBS (phosphate-buffered saline), *L. animalis* (10^6^ CFU/egg), *L. animalis* (10^7^ CFU/egg), *L. acidophilus* (10^6^ CFU/egg), *L. acidophilus* (10^7^ CFU/egg), *L. reuteri* (10^6^ CFU/egg), *L. reuteri* (10^7^ CFU/egg), *L. crispatus* (10^6^ CFU/egg), *L. crispatus* (10^7^ CFU/egg) and a mixture of the lactobacilli at the dose of 10^6^ CFU/egg and 10^7^ CFU/egg. The expression of the target genes was calculated relative to the reference gene (β-actin). Statistical significance among treatment groups was calculated using 1-way ANOVA followed by Tukey's comparison test. Error bars represent the standard error of the mean (SEM). The figures above the bars represent the number of birds expressing IL-17 within each respective group.

While the expression of IL-17 (a cytokine produced mainly by Th17) was observed in the bursa of only 2 birds of the untreated control group, lactobacilli treatment induced its expression in a larger number of birds, with *L. acidophilus* and *L. crispatus* at 10^6^ CFUs and *L. acidophilus, L. animalis*, and *L. reuteri* at 10^7^ CFUs induced it in 6 out of 8 inoculated birds (*P* ≥ 0.05) (Figure 6). Although the data did not subject to statistical analysis, the expression of IL-17 appears to be numerically higher in the groups treated with *L. acidophilus* (*P* ≥ 0.05).

## DISCUSSION

**Figure 7:**
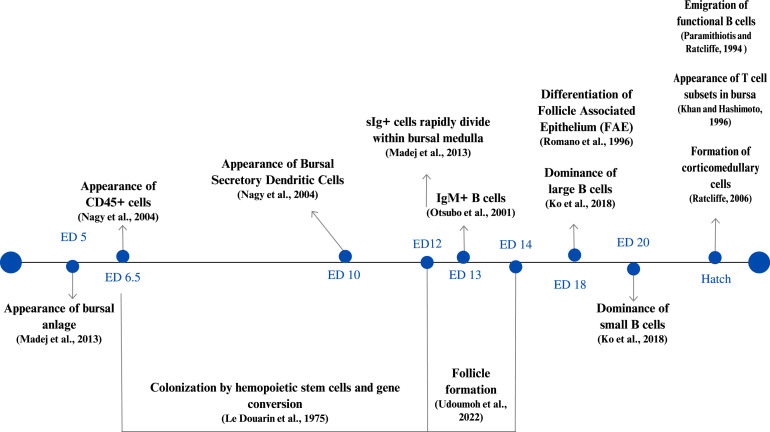
Cellular composition of the bursa of Fabricius during embryonic development (Khan and Hashimoto, 1996, Ko et al., 2018, Le Douarin et al., 1975, Madej et al., 2013, Nagy et al., 2004, Otsubo et al., 2001, Paramithiotis and Ratcliffe, 1994, Ratcliffe, 2006, Romano et al., 1996, Udoumoh et al., 2022).

With the poultry industry increasingly adopting *in ovo* vaccination as an alternative approach to post-hatch vaccination, there is a growing interest in early manipulation of the immune system of chick embryos, via feed additives, for enhancing vaccine immunogenicity and the immunocompetence of neonatal chickens. Among these feed additives, probiotics have demonstrated considerable promise in modulating immunity and improving the performance parameters of neonatal chicks when administered *in ovo* a few days before hatching (de Oliveira et al., 2014; Alizadeh et al., 2020, 2021, 2022; Shehata et al., 2021). While numerous studies have focused on the role of probiotics in modulating the innate and adaptive immune responses and in increasing the weight, morphological changes, and cellular composition of the lymphoid organs in hatched chicks (Cengiz et al., 2015; Hussein et al., 2020; Stefaniak et al., 2020), their impact on the development of lymphoid organs during the late embryonic stage is poorly understood. Of note is the bursa of Fabricius, a unique primary lymphoid organ in birds that plays an essential role in the ontogenetic development of adaptive immunity by generating a diverse antibody repertoire. The development of the bursa of Fabricius begins at ED5. It reaches its maximum size between 8 and 12 wk after hatching and remains functionally active until 6 months of age, after which it undergoes involution (Ko et al., 2018). The changes in the cellular composition of the bursa during embryonic development are depicted in Figure 7.

The distinct structure of the bursa provides a niche that supports B cell maturation and proliferation. Evidence indicates that bursal follicles predominantly constitute B lymphocytes (85–95%), followed by T lymphocytes (<5%), and other nonlymphoid cells (Palojoki et al., 1992; Kim et al., 2000), such as Bursal Secretory Dendritic Cells (**BSDCs**) and stromal cells (Mesenchymal Reticular Cells and macrophages) (Oláh et al., 2022). Since B and T cells are the major cell types in the bursa, this study was undertaken to investigate the potential of lactobacilli in altering the expression of genes involved in B development, differentiation and activation and genes expressed by other cellular populations (lymphoid and myeloid cells) during the early stage of bursal growth and development.

In general, our results revealed that *L. acidophilus* and the mixture of *Lactobacillus* species at the dose of 10^6^ CFU induced comparable gene expression levels of various cytokines produced by lymphoid and myeloid cells in the bursa of Fabricius, including IFN-γ, IL-12p40, IL-4, IL10, IL-13, IL-17, TGF-β, BAFF, and BAFF-R. Given the similarity in gene expression within these 2 particular groups (*L. acidophilus* and the mixture of *Lactobacillus* species), one could reasonably speculate that the observed effects might be related to the presence of *L. acidophilus* in the mixture rather than the combined effects of different lactobacilli.

It is also worth noting that the low level of gene expression observed in this study could be due to the fact that the bursal cells are not yet fully developed. While there is no evidence to support this hypothesis in the bursa, Abdul-Careem and colleagues have demonstrated developmental changes in cytokine gene expression in the spleen of developing chicken embryos and newly hatched chick. Their findings revealed that cytokine expression levels exhibited a gradual increase as the birds aged, with higher expression of IFN-γ, IL-4, IL-10, and IL-18 observed in the spleen of post-hatched chickens compared to chick embryos (Abdul-Careem et al., 2007). In another *in vitro* study, treatment of adult chicken splenic T cells with various mitogens resulted in cellular proliferation and production of IFN-γ and IL-2; however, no such effects were observed in the splenic T cells of day-old chicks, though they were phenotypically mature (Lowenthal et al., 1994). The lack of splenic T cell responsiveness in neonatal chicks implies that these cells have not fully developed their functional capabilities. In the context of bursal responsiveness, Alizadeh and colleagues observed that *in ovo* inoculation of a *Lactobacillus* mixture, including *L. salivarius, L. johnsonii, L. reuteri*, and *L. crispatus*, induced a higher cytokine expression in the bursa at 10 d of age, while little or no changes were observed at 5 d of age (Alizadeh et al., 2021). Taken together, the increased expression of genes associated with the development and functional maturity of bursal T and B cells highlights the potential immunostimulatory capacity of the *in ovo*-injected lactobacilli in enhancing the B cell maturation and immune responsiveness.

In the present study, the enhanced expression of IL-12p40, IL-17, and IFN-γ indicates that lactobacilli can promote the functional ability of lymphoid and myeloid cells in the bursa of Fabricius. This observation is in support of a previous study that found *in ovo* inoculation of a *Lactobacillus* mixture can augment the expression of these genes in newly hatched chicks (Alizadeh et al., 2021). While the excessive production of the proinflammatory IL-17 could potentially result in uncontrolled inflammation (Ge et al., 2020), it has been demonstrated that the controlled expression of IL-17 plays a crucial role in coordinating innate immunity against extracellular pathogens by promoting the expression of antimicrobial peptides (Lin et al., 2011; Brabec et al., 2023). It is therefore important to highlight that although probiotic lactobacilli induced the expression of IL-17 in a larger number of birds compared to the untreated control birds, the magnitude of its expression does not appear to be elevated to the level that would be detrimental to the host.

In addition to its role in promoting the differentiation of the naïve CD4+ cells to T regulatory (**Treg**) cells, TGF-β also plays a vital role in antibody diversification by driving the class-switching process toward IgA+ cells, the primary class of antibodies in mucosal secretions (Coffman et al., 1989). Indeed, Ko et al. (2018) have recently studied the phenotypic changes of the bursal B cells during embryonic development, and their result revealed that the percentage of IgA+ B cells was extremely low (<1%) during the embryonic stage and the first 2-days post-hatch (Ko et al., 2018). Hence, the notable induction of TGF-β expression in the bursa highlights the potential of the *in ovo*-delivered probiotic lactobacilli to provide stimulatory signals to bursal cells, leading to an increased diversity of the antibody repertoire.

Since B cell differentiation and proliferation in the bursa commence on ED15 and continue to proliferate until 8 to 12 wk of age (Oláh et al., 1986), it is tempting to speculate that early induction of genes responsible for B cell proliferation could lead to accelerated bursal development. IL-4 is known for its essential role in promoting the activation of mature B cells and immunoglobulin class switching to IgG1 and IgE (Yoshimoto, 2018). It is thought that the production of IL-13 might also promote the Th-2 response by suppressing the production of IL-12, an inhibitor of Th2 development (Leyva-Castillo et al., 2021). Furthermore, both IL-4 and IL-13 have been reported to promote the growth and proliferation of B cells (Junttila, 2018). In view of this, the lactobacilli-induced expression of IL-4 and IL-13 suggests that *in ovo* supplementation of lactobacilli during the late embryonic stage could accelerate B cell growth and proliferation and functional maturity of developing bursa.

B cell activating factor (**BAFF**) and B cell activating factor receptor (**BAFF-R**) are members of the tumor necrosis factor family and are known as key regulators of B cell survival, bursal cellularity, and phenotypic maturation (Ng et al., 2004). During the earliest embryonic stage (ED15–17), the bursa harbors a higher proportion of large than small B cell subpopulations; however, this ratio quickly reverses around E19 to 20, with small B cells becoming the predominant population (Ko et al., 2018). Given that large B cells are more proliferative and differentiated than small B cells, the induction of BAFF and BAFF-R expressions by lactobacilli suggests that supplementation of lactobacilli to chick embryos before ED19 could stimulate and extend the proliferative capacity of B cells.

In addition to their role in B cell activation and proliferation, evidence indicates that BAFF and BAFF-R represent the main prosurvival factors to naïve B cells by regulating apoptosis during the transitional B cell stages (Schneider, 2004). Since over 90% of the B cells undergo apoptosis during migration to secondary lymphoid organs after hatching (Lassila, 1989), the lactobacilli-induced expression of BAFF and BAFF-R could provide a survival signal to B cells during the transitional stages and migration process.

Overall, *in ovo* delivery of probiotics was associated with alterations in the expression of various immune system genes involved in the maturation of B and T cells and myeloid cells in the bursa of Fabricius of pre-hatched chicks. Nonetheless, more research is needed to further explore whether the observed changes in genes associated with cellular differentiation and proliferation resulted in any alterations to the phenotypic characteristics, cellular composition, and developmental trajectory of bursal cells.

In conclusion, the results of this study suggest that *in ovo* delivery of lactobacilli provides signals not only for B cell development and survival but also for promoting the immune function of other lymphoid and myeloid cells during bursal development. These findings highlight the potential of *in ovo* application of lactobacilli to promote bursal development.
